# Lateralization of gene expression in the honeybee brain during olfactory learning

**DOI:** 10.1038/srep34727

**Published:** 2016-10-05

**Authors:** Yu Guo, Zilong Wang, You Li, Guifeng Wei, Jiao Yuan, Yu Sun, Huan Wang, Qiuhong Qin, Zhijiang Zeng, Shaowu Zhang, Runsheng Chen

**Affiliations:** 1 Key Laboratory of RNA Biology, Institute of Biophysics, Chinese Academy of Sciences, Beijing 100101, China; 2 University of Chinese Academy of Sciences, Beijing 100049, China; 3 Honeybee Research Institute, Jiangxi Agricultural University, Nanchang, Jiangxi, 330045, China; 4 Research School of Biology, College of Medicine, Biology and Environment, The Australian National University, Australia; 5 Research Network of Computational Biology, RNCB, Beijing, 100101, China

## Abstract

In the last decade, it has been demonstrated that brain functional asymmetry occurs not only in vertebrates but also in invertebrates. However, the mechanisms underlying functional asymmetry remain unclear. In the present study, we trained honeybees of the same parentage and age, on the proboscis extension reflex (PER) paradigm with only one antenna in use. The comparisons of gene expression between the left and right hemispheres were carried out using high throughput sequencing. Our research revealed that gene expression in the honeybee brain is also asymmetric, with more genes having higher expression in the right hemisphere than the left hemisphere. Our studies show that during olfactory learning, the left hemisphere is more responsible for long term memory and the right hemisphere is more responsible for the learning and short term memory.

Lateralization of olfactory learning in the honeybee *Apis mellifera* has been demonstrated by Letzkus *et al*.^1^ and Rogers *et al*.^2^, who trained bees on the proboscis extension reflex (PER) paradigm with only one antenna in use^3^. The honeybee displayed a clear laterality in response to learned odors. Specifically, bees responded better to odors when they were trained through their right antenna^1^. Rogers *et al*. trained bees using both antennae, and found that at 1–2 hours after training memory recall was probable mainly via the right antenna, but from 6 hours after training a lateral shift occurred and the memory was recalled mainly via the left antenna^2^. The anatomy of the honeybee brain showed there were different types of neural connections between the mushroom body (MB) and other protocerebral areas of the honeybee’s brain. They were (1) unilateral neurons, with projection fields restricted to the ipsilateral protocerebrum; (2) recurrent neurons, which interconnect subcompartments of the MB, forming loops at different levels of the neuropil; and (3) bilateral neurons, which either interconnect both alpha-lobes or connect the ipsilateral alpha-lobe and protocerebral lobe with the dorsolateral protocerebral lobe of the contralateral hemisphere. It would seem that the right antenna and the associated neural structures form the basis for a short term and relatively temporary memory, and left antenna supports long-term memory, taking place from about 3 hours after training on^4^.

Left-right asymmetry of olfaction was also found in bumblebees, *Bombus terrestris*^5^. Lateralization had also been observed in visual learning of honeybees. By training honeybees on a modified version of a visual proboscis extension reflex task, it was found that bees learned a color stimulus better with their right eye^6^. Rogers’ team^7^ has shown that honeybees display a strong lateral preference for using their right antenna in social interactions. McNeill and Robinson^8^ found that an immediate early gene, *c-jun*, have an asymmetric expression in the honeybee brain during the PER paradigm. Other invertebrate species have also shown lateralization in olfactory learning, visual learning and other behaviors^9^,^10^,^11^,^12^,^13^,^14^. However, unlike in vertebrates, the asymmetries of invertebrate brains have not yet been well studied^2^.

The olfactory sensors of *Apis mellifera* have been shown to possess significant differences between the right and left antenna^1^. These differences might contribute to the lateralization of the olfactory learning, but it is unclear whether a whole genome study of gene expression would also show asymmetry between the two hemispheres of the honeybee brain. To further study the possible asymmetry of the honeybee brain in learning and memory, we trained honeybees on the PER paradigm, and determined the expression levels of mRNAs and microRNAs in the two brain hemispheres with high throughput sequencing^15^.

To minimize the effects of the genetic background, we used a single drone inseminated (SDI) queen to set up the colony, which ensured a higher genetic similarity among the workers. To further minimize the differences between individuals, we used a special technique developed by Zhang *et al*.^1^ at the Australian National University, by which we were able to train the left and right hemispheres of each individual bee separately, and then make a comparison between the two hemispheres of the same bees.

## Results

### Right antenna training induces higher learning and memory ability

**Figure 1:**
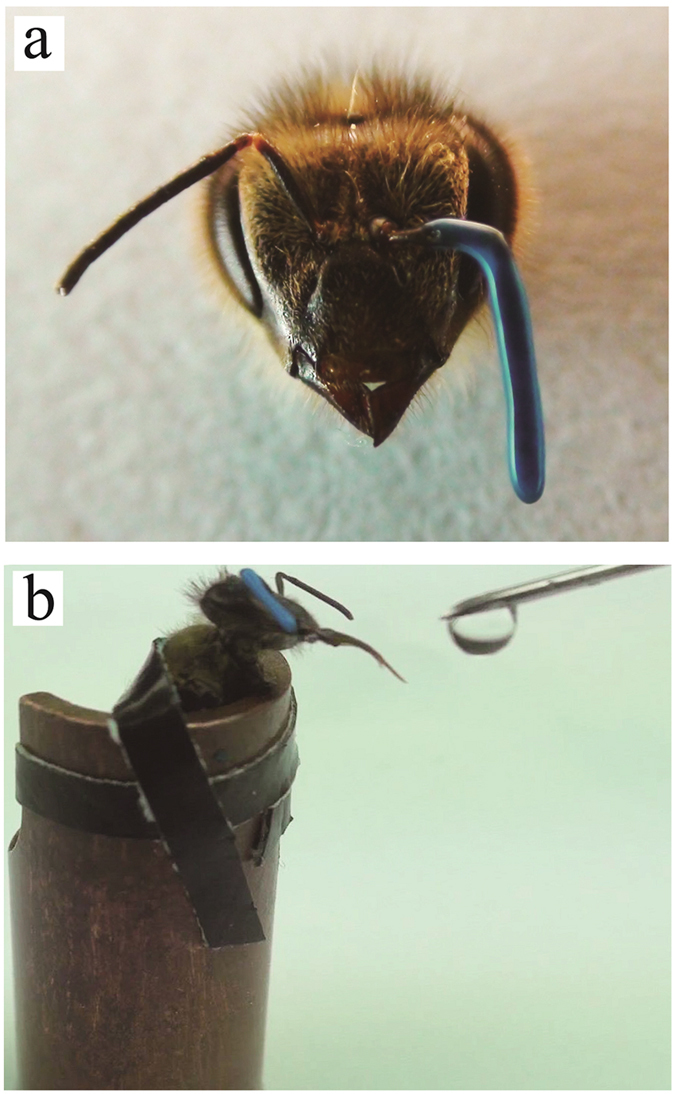
PER experiment. (**a**) Antenna covered with silicone compound. (**b**) Feeding the bees with sugar water.

The PER paradigm was performed on the honeybees with one of their antenna covered by a silicone compound. The experiment was carried out on three groups. One group had their left antenna covered (LAC), the second group had their right antenna covered (RAC), and a third group with both of their antennae left uncovered constituted the control (Control), and was not trained with the PER paradigm (Fig. 1a). For the two antenna covered groups and the control group, we have done several independent experiments. Each time, for the LAC and RAC group, we trained about 90 workers, about 20 bees passed the memory recall test and were sampled. Finally, heads of 112 bees in the LAC group and 104 in the RAC group were sampled. The control groups were also sampled from several independent experiments, and 140 untrained bees were sampled. These sampled bees were divided into two biological replicates for total RNA extraction.

Lemon + sugar water was used as the positive stimulus (reward), while vanilla + salt water was used as the negative stimulus (punishment) to perform the PER paradigm on the bees (Fig. 1b). In the retention test, bees giving a correct response to the positive stimulus and not giving a response to the negative stimulus were considered as having learned the two stimuli and passed the test. Bees which passed the test were dissected to obtain the two hemispheres of their brains. The left and right hemispheres were dealt with separately in the high throughput sequencing process.

During the training, we found that the ratio of bees passing the retention test to the number of bees alive at 24 hours was significantly different between the LAC and RAC group (p value <0.1, by the chi-square test) (Table 1). Bees in the LAC group were better at memory retention. This result was consistent with the previous study of Letzkus *et al*.^1^ but not with that of Rogers and Vallortigara *et al*.^2^. The difference is that Letzkus *et al*. and we covered one antenna before training and kept the antenna covered during testing at 24 hours, whereas Rogers and Vallortigara trained bees without covering the antennae and covered one antenna only before testing recall at 1–2 hours or 24 hours. This latter procedure showed a shift from right to left antenna in the recall of the memory. Hence, it is only when the bee is forced to learn using one antenna and asked to recall using that same antenna that the left antenna seems to have no role in the memory recall.

### Gene expression in the two hemispheres is asymmetric

**Figure 2:**
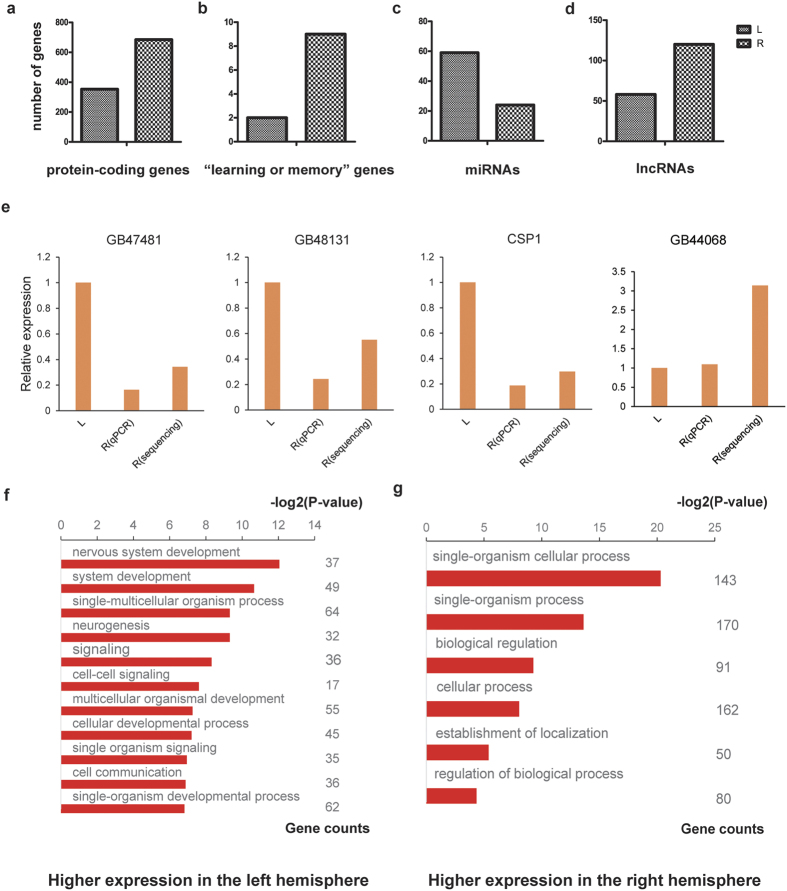
Gene expression between the two brain hemispheres in the Control group. (**a**–**d**) Number of genes with more than 2-fold change in expression between left and right brain hemispheres. (**a**) Protein-coding genes, (**b**) “Learning or memory” genes, (**c**) microRNAs and, (**d**) lncRNAs. (**e**) qRT-PCR analysis of four genes. L Expression (sequencing or qRT-PCR) in the left hemisphere (“L”) of the control group is set to one. “R (qPCR)” denotes the result of qRT-PCR in the right hemisphere of the control group, and “R (sequence)” denotes the result of high throughput sequencing in the right hemisphere of the control group. (**f–g**) GO analysis of genes with higher expression in (**f**) the left and (**g**) the right brain hemisphere, respectively, of the control group. The X axis is the negative of the log2 of the corrected p-value.

We define a differentially expressed protein-coding gene (DEG) as a gene that shows more than 2-fold change (p-value <0.05) in expression between two compared states or conditions. We first examined the expression levels of all protein-coding genes in the control group, and found 1038 DEGs between the two brain hemispheres^16^,^17^, of which 353 had higher expression in the left hemisphere, and 685 had higher expression in the right hemisphere (Fig. 2a). Thus, among the DEGs considerably more genes showed the highest expression in the right hemisphere.

In order to validate these results, quantitative reverse transcription PCR (qRT-PCR) were performed on 4 of the DEGs in the control group. The results of the qRT-PCRs confirmed the results obtained by high throughput sequencing (Fig. 2e).

We next tried to obtain an indication of to what extent the control group DEGs might be involved in learning and memory tasks. To this end we retrieved the sequences of all 2528 genes annotated with the term “Learning or memory” in the Gene Ontology (GO) database. Gene Ontology is the framework for the model of biology. The GO defines terms used to describe gene function, and relationships between these concepts. By sequence alignment, we found 320 honeybee genes that are homologues of these learning or memory associated genes, and defined these as “learning or memory” genes. Among the control group DEGs, there were 11 “learning or memory” genes, 9 and 2 being more highly expressed in the right and left hemispheres, respectively (Fig. 2b). One of these was acetylcholinesterase 1(*AChE-1*), which is a key enzyme in cholinergic nerve conduction and was significantly up-regulated in the right hemisphere. *AChE-1* can rapidly hydrolyze the neurotransmitter acetylcholine to terminate cholinergic neurotransmission and maintain the sensitivity of nerve impulse conduction^18^.

In accordance with the above, we further defined a differentially expressed microRNA (DE-miRNA) as a microRNA that showing more than 2-fold change in expression. A comparison of the microRNA expression in the control group showed that, of the 222 microRNA genes that have been annotated in *Apis mellifera*^19^,^20^, 83 were identified as differentially expressed between the two brain hemispheres. Of these, 24 and 59 showed the highest expression in right and left hemispheres, respectively, thus presenting an overall picture opposite to that found for the mRNAs (Fig. 2c).

The miRanda software^21^,^22^,^23^ was used to predict mRNA targets of DE-miRNAs. The result showed that among the targets of DE-miRNAs with lower expression in the left hemisphere, there were 28 DEGs with higher expression in this hemisphere, and similarly among the targets of DE-miRNAs with lower expression in the right hemisphere, there were 143 DEGs with higher expression in this hemisphere.

The data also allowed for a comparison of the expression of long noncoding RNAs (lncRNAs) in the honeybee brain. Analysis of the control group data showed that 467 of the 3999 annotated lncRNAs in the honeybee genome^24^ were expressed in the brain. Of these, 178 lncRNAs had a more than 2-fold change between the two brain hemispheres, 58 with higher expression in the left hemisphere, and 120 with higher expression in the right hemisphere (Fig. 2d).

### Gene Ontology analysis of DEGs in the control group

Using the ‘reciprocal best hit’ method^25^,^26^, we identified all genes in the honeybee genome that had a possible fruit fly orthologue. Of the 353 and 685 DEGs that were up-regulated in the left and right brain hemispheres, respectively, of the control group, 149 and 217 genes had possible orthologues in the fruit fly.

GO analysis was performed on these genes. The results showed that an enrichment of genes annotated with “system development”, especially “nervous system development”, and “signaling” among the DEGs with higher expression in the left hemisphere, while among DEGs with higher expression in the right there were more genes annotated with “biological regulation” and “regulation of biological process” (Fig. 2f,g)^27^,^28^.

### Whole brain expression of protein-coding genes after training

We next moved the analysis to gene expression in the brains of the trained bees. In order to first obtain an estimate of whole brain gene expression, we took the average FPKM of each gene in the left and right brain hemispheres of bees in the same training group as the whole brain expression of this gene in this group. When comparing to the control group, there were 3080 DEGs in the LAC group and 1293 DEGs in the RAC group. In the LAC group, the number of up-regulated DEGs (relative to control brains) was higher than the number of down-regulated DEGs while the opposite was the case in the RAC group (Table 2).

### Changes in gene expression are significantly different in the two hemispheres

**Figure 3:**
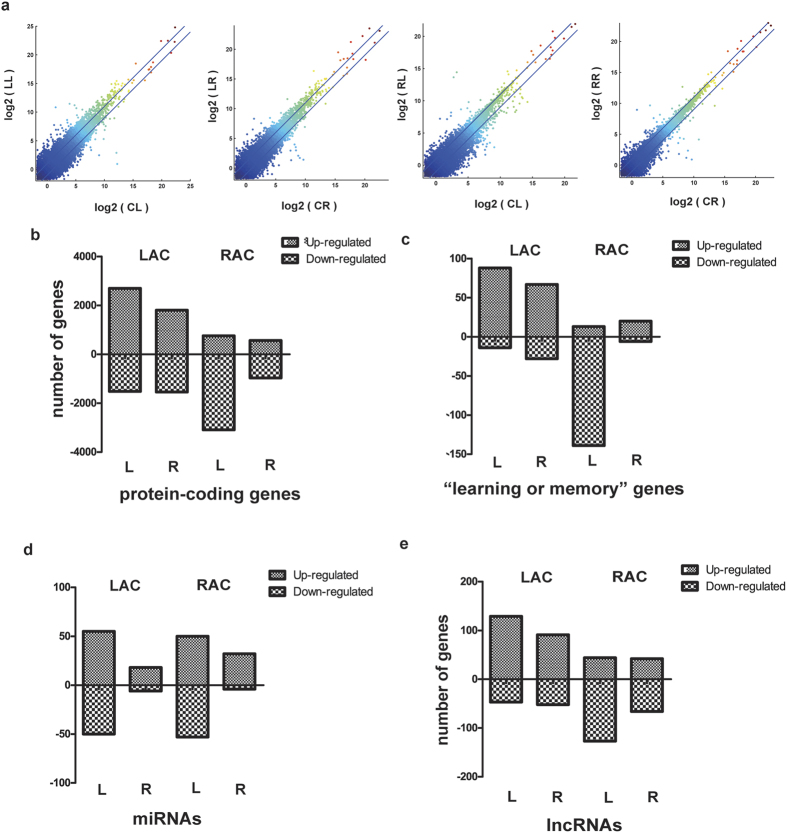
Gene expression pattern during olfactory learning. (**a**) Identification of DEGs in each brain hemisphere of trained bees. Each panel is a plot of the expression of all genes in a given hemisphere (Y-axis) of a trained group against the expression of the same genes in the same hemisphere in the control group (X-axis). Expression is represented as the log_2_ of the FPKM. Genes falling above or below the parallel lines were taken as up- and down-regulated DEGs, respectively, of that particular brain hemisphere. The first letter in the abbreviations denotes the control (C−), LAC (L−) and RAC (R−) groups, and the second letter denotes the left (−L) and right (−R) hemispheres, respectively. (I.e., CL is the left hemisphere of the control group, and so forth). (**b**–**e**) Different patterns of DEGs in the brain hemispheres of the RAC and LAC groups. The panels show the number of up- and down-regulated DEGs in each brain hemisphere of bees from the RAC and LAC groups. L and R denote left and right brain hemisphere, respectively.

We subsequently examined the changes in gene expression between the brain hemispheres within the two trained groups. For each group, the gene expression in each brain hemisphere was compared to the corresponding brain hemisphere of the control group (i.e, the left brain hemisphere of the LAC group was compared to the left brain hemisphere of the control group, and so forth). Genes in each brain hemisphere showing more than a 2-fold change in gene expression relative to the respective hemisphere of the control group were thus regarded as differentially expressed genes (DEGs) (Fig. 3a).

Compared in this manner, the left and right hemispheres of the LAC group had 4214 and 3345 DEGs, respectively. In the left hemisphere of the LAC group, 2697 DEGs were up-regulated relative to the left hemisphere of the control group, and 1517 DEGs were down-regulated. The corresponding figures for the right hemisphere of the LAC group were 1804 up-regulated DEGs and 1541 down-regulated DEGs relative to the right hemisphere of the control group. For both brain hemispheres, the number of genes with increased expression after training was thus higher than the number of genes with reduced expression levels.

The same comparison between the RAC group and the control group showed that there were 3847 DEGs between the left hemisphere of the RAC group and the left hemisphere of the control group. The majority of these genes (3091) were down-regulated in the RAC group relative to the control, whereas 756 were up-regulated. In the right hemisphere of the RAC group, there were 1533 DEGs when compared to the right hemisphere of the control group, and also for this hemisphere there were more down-regulated (967) than up-regulated (566) genes. Thus, for the RAC group, there was a higher number of genes with reduced than with increased expression after training in both hemispheres (Fig. 3b).

We also made a similar comparison between the hemispheres of trained and control groups for the 320 “learning or memory” genes. The results resembled those obtained for all protein-coding genes, with the exception that for the right hemisphere of the RAC group the number of up-regulated genes was higher than the number of down-regulated genes (Fig. 3c). However, as the number of DEGs within this selection of genes was not large, we are uncertain as to the significance of this observation.

When microRNA expression was examined in the same way, there were notable differences compared to the results obtained for protein-coding genes. For both the LAC and RAC groups, the number of DE-miRNAs relative to the same hemispheres of control brains, was significantly higher in the left than the right hemisphere. The number of DE-miRNAs in the left hemispheres of the LAC and RAC groups were 105 and 103, respectively (Fig. 3d), and of these, 81 were the same in both groups. The numbers of up-regulated and down-regulated microRNAs in the left hemisphere were about the same. In the right hemispheres of both groups, the number of DE-microRNAs were lower, 24 and 36 for the LAC and RAC groups, respectively, and the numbers of up-regulated DE-microRNAs relative to control were considerably higher than the numbers of down-regulated DE-miRNAs.

The same comparison between brain hemispheres of trained and control bees carried out for the lncRNAs returned trends similar to those found for protein-coding genes (Fig. 3e) (Table S1).

### Many DEGs are predicted targets of DE-miRNAs in LAC and RAC groups

We predicted targets of the DE-miRNAs in the LAC and RAC groups, and compared the predicted target genes with DEGs having opposite expression in the same group (Table 3). The result showed that many DEGs were predicted to be a target of DE-miRNAs, overall, these target DEGs showed a similar expression pattern in the two hemispheres to that of the protein-coding genes, indicating that regulation of microRNA might be one of the main reason causing asymmetric expression of protein-coding genes in the two hemispheres.

Of the 320 “learning or memory” genes, 207 were predicted to be a target of at least one DE-miRNA. The ratio of differentially expressed “learning or memory” genes to the target of corresponding DE-miRNAs are even higher (Table 4), indicating that learning and memory process in honeybee is widely regulated by microRNAs.

Some particular genes reported to play important roles in signal transduction pathways, were found both as DEGs and as a target of the DE-miRNAs. These included as *Tyr1* (tyrosine 1), GABA receptor β, *nAChRb1* (nicotinic acetylcholine receptor beta1 subunit), muscarinic acetylcholine receptor, *5-HT2 β* (5-hydroxytryptamine receptor beta), and octopamine receptor 1 (Table S2).

## Discussion

Increasing evidence suggests that brain lateralization is not only a vertebrate phenomenon, but also occurs in invertebrates^29^,^30^. In the honeybee, lateralization of olfactory learning, visual learning and social behavior have been demonstrated^1^,^6^,^7^. The mechanism underlying the lateralization is not clear, and we consequently designed the reported experiment to investigate the molecular correlates of bee brain lateralization.

Our study is the first to investigate differences in gene expression between the two brain hemispheres of the honeybee. We found that about 6% of the protein-coding genes have a more than 2-fold change in expression level between the two hemispheres, and that a higher number of the differentially expressed genes were up-regulated in the right brain hemisphere.

In contrast, there were more microRNAs having higher expression in the left hemisphere. The commonly established function of microRNAs is that of suppressors of protein-coding genes, and it may therefore be unsurprising that most up-regulated DE-miRNAs were found in the brain hemisphere with most down-regulated protein-coding DEGs, and vice versa.

The observed differences in gene expression between the two hemispheres indicate that there must be sort of relationship between lateralization of behavior and the gene expression in the brain. Previous studies have shown that the overall gene expression level of the whole brain is down-regulated after learning and memory^25^,^31^,^32^. This down-regulation is thought to be caused by the formation of long term potentiation (LTP)^25^. In vertebrates, such as mice, it has been reported that LTP takes place more in the left hemisphere than the right hemisphere^33^. In our experiment, only the RAC group showed overall down-regulation of gene expression (indicative of LTP) at the whole brain level when the bees were trained on an olfactory associative task with only one antenna in use. When the brain hemispheres were considered separately, this down-regulation was only in the RAC group, especially in the left hemisphere. It is thus possible that in the honeybee, long term potentiation is more restricted to the left hemisphere during olfactory learning.

The down-regulation of protein-coding genes in LTP is thought to be partially mediated via time-dependent regulation of microRNA expression^25^,^33^. In our experiment, we found that many DEGs, especially “learning or memory” genes, were predicted to be the target of corresponding DE-miRNAs, which suggested that microRNAs were involved in learning and memory. The fact that there were more microRNAs having higher expression and more DE-miRNAs in the left than in the right hemisphere indicates that the influence of microRNAs may mainly exist in the left hemisphere.

Rogers and Vallortigara found a shift in olfactory memory recall from the right antenna to the left antenna, and they also assumed that the long-term memory is more associated with the left hemisphere^2^. In their experiment, bees trained with the right antenna showed better performance only when the memory recall was done 1 h after training, which they took to indicate that the right hemisphere is better at learning and short term memory. In contrast to our experiments, in which training and memory recall test were completed with the corresponding antenna covered, both antenna were left uncovered during the whole paradigm in their experiment. As memory acquisition is a prerequisite for LTP, the preference in the right hemisphere for memory acquisition takes precedence over the preference in the left hemisphere of the LTP, thus, the final result obtained in our experiment, even though our memory recall test were done fully 24 h after training, was that training with the right antenna produced better memory retention.

The left and right hemispheres of the human brain are different in function^34^,^35^, and the same phenomenon has been reported in many higher animals^29^,^30^. Our experiments support the existence of functional differences between the brain hemispheres in *Apis mellifera*, and suggest that the lateralization of honeybee behavior may be a reflection of the gene expression patterns in the brain hemispheres. Specifically, we found that in the process of learning or memory, the gene expression patterns of the two hemispheres were quite different. We hypothesize that during olfactory learning, there is a functional division between the two brain hemispheres, the left hemisphere being more responsible for long term memory, and the right hemisphere being more responsible for the learning and short term memory. We will in the future carry out further in-depth studies, hoping to elucidate the molecular mechanisms of this phenomenon in more detail.

## Materials and Methods

### Experimental bees

The *Apis mellifera* worker bees used in this study were sampled from the Honeybee Research Institute, Jiangxi Agricultural University, China (28.46°N, 115.49°E), according to standard beekeeping techniques. In order to ensure a highly similar genetic background among the workers and reduce the variation caused by individual differences, one colony headed by an artificially single drone inseminated (SDI) queen was set up. To ensure that the bees used in our experiment were of the same age, bee combs containing several hundred prepupae were packaged in a nylon net in the evening. The next morning the newly emerged bees were gathered into rectangular boxes, and then maintained in an incubator at a constant temperature of 34 °C in a light-dark cycle of 12 hours light and 12 hours dark. The bees were fed with 1 M sucrose and pollen three times daily. After one week, they were removed from the box to be used for experiments.

### PER experiment

The PER experiment began on the morning of the eighth day after the emergence of the worker bees. The experimental procedure closely followed those of Letzkus^1^ and one of our previous studies^31^. Details are provided in the Supplementary Materials and Methods.

### Brain dissection

The brains were dissected from the sampled honeybee heads using a sharp razor blade in normal saline (137 mmol/L NaCl, 2.7 mmol/L KCl, 10 mmol/L Na2HPO4, 2 mmol/L KH2PO4) in a 4 °C environment. Immediately after dissection, the brain was divided into left and right halves along the mid axis of the mushroom bodies, and each brain half was collected into a 1.5 ml RNase-free eppendorf tube on dry ice, and subsequently stored at −80 °C until further use.

### RNA isolation and cDNA library preparation for Illumina Solexa sequencing

For each group, the sampled heads were randomly divided into two portions as two biological replicates, then the brain tissues were dissected for RNA isolation. For each biological replicate, both total RNA and microRNA were isolated. Total RNA was isolated using the Invitrogen Trizol kit. MicroRNA libraries were constructed using the Illumina TruSeq small RNA sample preparation kit, and mRNA libraries were constructed using the Illumina mRNA-Seq 8 sample kit. The sequencing process was carried out on the Illumina Hiseq2000 platform, and the raw data was collected according to the standard Illumina Solexa pipeline.

### Sequencing Data analysis

The mRNA-seq data was treated using the cufflinks v2.2.1 workflow^36^. First, we aligned the reads with TopHat to the *Apis mellifera* genome, assembly Amel_4.5. Second, aligned transcripts were assembled with Cufflinks, then the transcripts were merged to the final transcriptome assembly using the Official Gene Set OGSv3.2 as a reference genome annotation, and finally cuffdiff was performed to calculate the FPKM (fragments per kilobase per million mapped reads).

As to potential miRNAs, we removed the adaptors as the first step. Reads of 18–30 nt length were kept and compared with the microRNA data of *Apis mellifera* in the mirBASE (http://www.mirbase.org) by blast. The RPM (reads per million) were calculated as a measure of the expression levels of the microRNAs.

### Validation of high throughput sequencing data results by qRT-PCR

The honeybees were sampled from another SDI colony, and their brains were dissected and divided into left and right halves according to the above method. Total RNAs were isolated from these brain tissues using the Invitrogen Trizol kit. The SuperScript III reverse transcriptase was employed to synthesize the first-strand cDNA. qPCR analysis was performed with 0.5 μL cDNA as template in a 20 μL reaction volumes using SYBR green master mixture on the Rotor-Gene^®^ Q real-time cycler (Qiagen). Ribosomal protein L8(RPL8) was chosen as an internal control. The expression levels of each gene were normalized to RPL8 and relative expression was calculated by the delta-delta Ct method. The primer sequences are provided in Supplementary Materials and Methods.

### Homologous genes analysis

Putative orthologous genes between fruit fly and honeybee were identified using the “reciprocal best hit” method^26^. The learning or memory genes were compared with the list of all genes annotated with the GO term “learning or memory” using blastp directly between the peptide sequences. Homologous lncRNAs were identified by blast against the Noncode database 4.0(http://www.noncode.org). Functional predictions for the homologous lncRNAs were also obtained from the Noncode database 4.0.

### microRNA target prediction

miRanda was used to predict targets of the honeybee microRNAs. Potential targets with a max score of more than 155 were selected for further analysis.

## Additional Information

**How to cite this article**: Guo, Y. *et al*. Lateralization of gene expression in the honeybee brain during olfactory learning. *Sci. Rep*. **6**, 34727; doi: 10.1038/srep34727 (2016).

## Supplementary Material

Supplementary Information

## Figures and Tables

**Table 1:** PER training result.

	Trained	Survival	Learned	Learned/Survival
LAC	442	346	102	28.99%
RAC	554	458	104	22.71%
Control	total 140			

**Table 2:** Changes in whole brain gene expression.

	LAC	RAC
Total	3080	1293
Up	1975	333
Down	1105	960

The table shows the number of genes whose average expression across both brain hemispheres in the LAC or RAC group is 2-fold higher (up) or lower (down) than the average expression across both brain hemispheres in the control group.

**Table 3:** Comparison between DEGs and the target of the DE-miRNAs.

	LL up	LL down	LR up	LR down	RL up	RL down	RR up	RR down
DE-miRNAs	55	50	18	6	50	53	32	4
All predicted targets	2946	5056	3107	619	2142	5504	5176	236
Corresponding DEGs	1517	2697	1541	1804	3091	756	967	566
Shared	243	987	380	99	527	210	117	7
Percentage	16.02%	36.60%	24.66%	5.49%	17.05%	27.78%	12.10%	1.24%

The first letter in the abbreviations denotes the control (C−), LAC (L−) and RAC (R−) groups, and the second letter denotes the left (−L) and right (−R) hemispheres, respectively. (I.e., CL is the left hemisphere of the control group, and so forth), “up” or “down” stand for up-regulated or down-regulated DE-miRNAs. The “corresponding DEGs” are DEGs that show “opposite” expression to the DE-miRNAs (i.e. are up-regulated when the DE- miRNA is downregulated, and so forth) in the same sample. “Shared” is the number of “corresponding DEGs” that are also predicted targets of the miRNAs in the same column.

**Table 4:** Comparison between “learning or memory” genes and the target of the DE-miRNAs.

	LL up	LL down	LR up	LR down	RL up	RL down	RR up	RR down
DE-miRNAs	55	50	18	6	50	53	32	4
All predicted targets	2946	5056	3107	619	2142	5504	5176	236
Corresponding “learning or memory” genes	14	88	28	67	140	14	6	20
Shared	7	47	9	11	48	5	2	1
Percentage	50.00%	53.41%	32.14%	16.42%	34.29%	35.71%	33.33%	5.00%

The corresponding “learning or memory” genes is the “learning or memory” genes having the opposite regulation in the same samples.
