# Supplementary material for: Lateralization of gene expression in the honeybee brain during olfactory learning
Source: Sci Rep. 2016 Oct 5;6:34727. doi: 10.1038/srep34727 (PMC5050455; doi:10.1038/srep34727)
Supplement: Supplementary Information [file srep34727-s1.doc]

**Lateralization of gene expression in the honeybee brain**

**during** **olfactory learning**

**Yu Guo^1,2,+^, Zilong Wang^3,+^,You Li^3^,Guifeng Wei^1,2^,Jiao Yuan^1,2^,Yu Sun^1,2^, Huan Wang^3^, Qiuhong Qin^3^, Zhijiang Zeng^3^, Shaowu Zhang^1,3,4,*^ & Runsheng Chen^1,5,*^**

^1^CAS Key Laboratory of RNA Biology, Institute of Biophysics, Chinese Academy of Sciences,

Beijing 100101, China;

^2^University of Chinese Academy of Sciences, Beijing 100049, China;

^3^Honeybee Research Institute, Jiangxi Agricultural University, Nanchang, Jiangxi, 330045, China;

^4^Research School of Biology, College of Medicine, Biology and Environment, The Australian National University, Australia;

^5^Research Network of Computational Biology, RNCB. Beijing, 100101, China.

^*^correspondence and requests for materials should be addressed to Z.Z. ([bees1965@sina.com](mailto:bees1965@sina.com)) or S.Z. ([shaowu.zhang@anu.edu.au](mailto:shaowu.zhang@anu.edu.au)) or R.C. ([crs@sun5.ibp.ac.cn](mailto:crs@sun5.ibp.ac.cn)).

^+^these authors contributed equally to this work.

**Supplementary Materials and Methods**

**PER experiment.** The PER experiment began on the morning of the eighth day after emergence of the worker bees. The bees were removed from the rectangular boxes and randomly assigned into three groups, two trained groups (LAC and RAC) and one untrained group (control). The bees were briefly cooled on ice for about 5 min until they stopped moving. Then, each bee was fixed in a metal tube with thin strips of tape so that only the two forelegs and head were free to move. For the two trained groups, one of their antennae was covered with a two-component silicone compound to ensure that the bees were trained by a single antenna, whereas in the control group both antennae were left free. Thereafter, all bees were briefly fed with one to two drops of 1 M sugar solution and moved back to the incubator to recover.

Two hours later, the bees were trained with a positive stimulus and a negative stimulus. In this experiment, lemon odor plus 1M sucrose solution was used as the positive stimulus (reward) and vanilla odor plus saturated saline was used as the negative stimulus (punishment). To prepare these two stimuli, 30 µl lemon and vanilla essence were dissolved in 4 ml 1M sucrose solution and saturated saline, respectively. The bees were trained to discriminate between these two different odors. During training, a small suction fan connected with a plastic pipe was placed behind the bees to maintain a constant flow of odor during giving the stimulus and to quickly remove any residual odor traces before beginning the training of the next bee. For each bee, three trials with an interval of 6 mins were given. Each trial contained a positive and a negative stimulus training. On the first trial, the bee was given a positive conditioned stimulus. A droplet was placed about 1-2 cm from the bee’s antennae using a 2.5 ml syringe with needle until the bee extended its proboscis and tasted the solution. If the bee did not extend its proboscis after 5 s, we briefly touched the antennae with the stimulus drop to prompt it to give a response to the odor. Then, the bee was given the negative stimulus with the same procedure, touching the antennae at the end of this 5 s period if the bee did not extend its proboscis. When the bee extends its proboscis it will receive a punishment caused by the salt solution. Bees that never extended their proboscis or were inactive were discarded after the first trial. After training, the bees were fed with 2-3 drops of 1M sugar solution and returned to the incubator for overnight storage.

Retention tests were carried out in the morning of the next day after the training. For the LAC and RAC group, the silicone were not removed until the retention tests were finished. At this time, the bees were first given the negative stimulus, and then the positive stimulus. As in the training, the stimulus droplet was placed over the antennae of the bee at a distance of 1-2 cm for 5 s, without touching the antennae. If the bee extended its proboscis within 5 s when it was given a positive stimulus but not when given the negative stimulus, it was regarded as correct, otherwise wrong. Each bee was tested in three trials with an interval of 6 mins. Bees giving a correct response to the positive stimulus in at least two of the three trials and not giving a response to the negative stimulus in all the three trials were considered as having learned the two stimuli and passed the test. After completion of the tests, the heads of the trained bees that had passed the test and those of the untrained bees (control group) were sampled and stored in liquid nitrogen. In total, heads of 112 bees in the LAC group, 104 bees in the RAC group and 140 untrained bees were sampled.

**Primers for qRT-PCR.** The following quantitative RT-PCR primers were used: XLOC_000981, 5’- TCCTAAGTTAAATTCGCAACAACA-3’ and 5’- TGAATCTTTTTACTTGCTC-

TTGACA-3’; XLOC_012916, 5’- CGTTCGAGAGTATCGTGCAA-3’ and 5’- GATGTTAGGCGTGGCAGATT-3’; XLOC_014008, 5’- ATCGATGCAGGCTCTTGTTT-3’and 5’TCTTATGCGCCTTCGTCTTT-3’; XLOC_011655, 5’- AGATGGCGGGAAATAAGGTC-3’ and 5’- TTTTCCCCCTTTAAAAGATTCA-3’; and RPL8, 5’- CTCAAAGCTGGTCGTGCATA-3’ and 5’- TGGATGTTCAACAGGGTTCA-3’.

**Table S1 Up- and down-regulated DEGs in the two brain hemispheres of the RAC and LAC groups**

**Coding gene**s

|  | **LAC** |  | **RAC** |  |
| --- | --- | --- | --- | --- |
|  | **L** | **R** | **L** | **R** |
| Total | 4214 | 3345 | 3847 | 1533 |
| Up | 2697 | 1804 | 756 | 566 |
| Down | 1517 | 1541 | 3091 | 967 |

**"Learning or memory" gene**s

|  | **LAC** |  | **RAC** |  |
| --- | --- | --- | --- | --- |
|  | **L** | **R** | **L** | **R** |
| Total | 102 | 95 | 152 | 26 |
| Up | 88 | 67 | 13 | 20 |
| Down | 14 | 28 | 139 | 6 |

miRNAs

|  | **LAC** |  | **RAC** |  |
| --- | --- | --- | --- | --- |
|  | **L** | **R** | **L** | **R** |
| Total | 105 | 24 | 103 | 36 |
| Up | 55 | 18 | 50 | 32 |
| Down | 50 | 6 | 53 | 4 |

lncRNAs

|  | **LAC** |  | **RAC** |  |
| --- | --- | --- | --- | --- |
|  | **L** | **R** | **L** | **R** |
| Total | 176 | 143 | 171 | 108 |
| Up | 129 | 91 | 44 | 42 |
| Down | 47 | 52 | 127 | 66 |

**Table S2 Important genes in signal transduction pathways, which are both DEGs and the target of DE-miRNAs.** “1” means this gene is both DEGs and the target of DE-miRNAs, “0” means this gene is only DEGs or the target of DE-miRNAs, “-” means this gene is neither DEGs nor the target of DE-miRNAs.

|  | LL up | LL down | LR up | LR down | RL up | RL down | RR up | RR down |
| --- | --- | --- | --- | --- | --- | --- | --- | --- |
| muscarinic acetylcholine receptor | 0 | 0 | 0 | - | 1 | 0 | 0 | - |
| octopamine receptor beta-3R | - | - | 0 | - | 1 | - | - | - |
| octopamine receptor1 | - | 1 | 0 | - | - | 0 | 0 | - |
| nAChRb1 | 0 | 1 | 0 | - | 0 | 0 | 0 | - |
| GABA receptor beta | 0 | 0 | - | - | 1 | 0 | 0 | - |
| Adenylate cyclase 5 | 0 | 0 | 0 | - | 1 | 0 | 0 | - |
| Adenylate cyclase 9 | 0 | - | - | - | 1 | - | 0 | - |
| kainate 2C | 0 | 1 | - | - | 0 | 0 | - | - |
| kainate 2A | 0 | 0 | 0 | - | 1 | 0 | 0 | - |
| alpha-2B adrenergic receptor | 0 | 0 | - | - | 1 | 0 | 0 | 0 |
| 5-HT2 beta | 1 | - | - | - | 1 | 0 | - | - |
| kainate 1 | 0 | 1 | 0 | - | 1 | 0 | - | - |
| kainate 2B | - | 1 | 0 | - | 1 | 0 | 0 | - |
| mGluR4 | 0 | 1 | 0 | - | 1 | 0 | 0 | - |
| Tyr1 | 1 | - | - | - | 1 | 0 | - | - |
